# Supplementary figures and images for: Wolbachia-mediated resistance to Zika virus infection in Aedes aegypti is dominated by diverse transcriptional regulation and weak evolutionary pressures
Source: PLoS Negl Trop Dis. 2023 Oct 2;17(10):e0011674. doi: 10.1371/journal.pntd.0011674 (PMC10569609; doi:10.1371/journal.pntd.0011674)

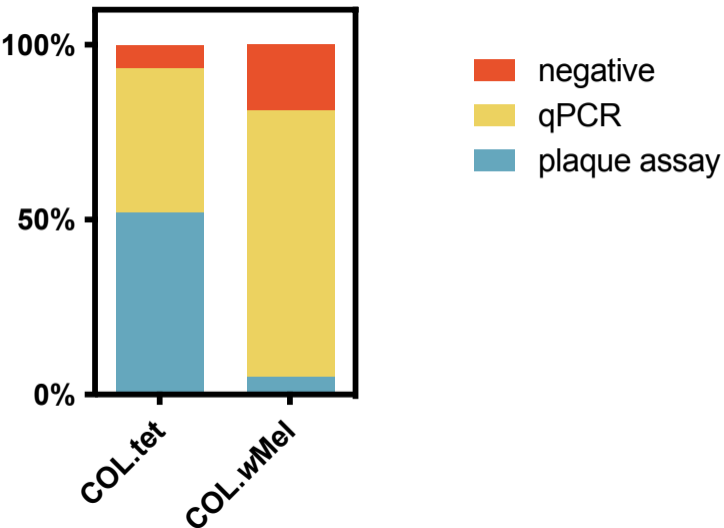

Supplement: S1 Fig — Infection prevalence was measured by combining the number of PFU-positive mosquito samples via plaque assay (blue) and vRNA-positive samples via qPCR (yellow). Overall infection prevalence was 81% for COL.wMel and 93% for COL.tet. (PDF) [file pntd.0011674.s001.pdf]

A.

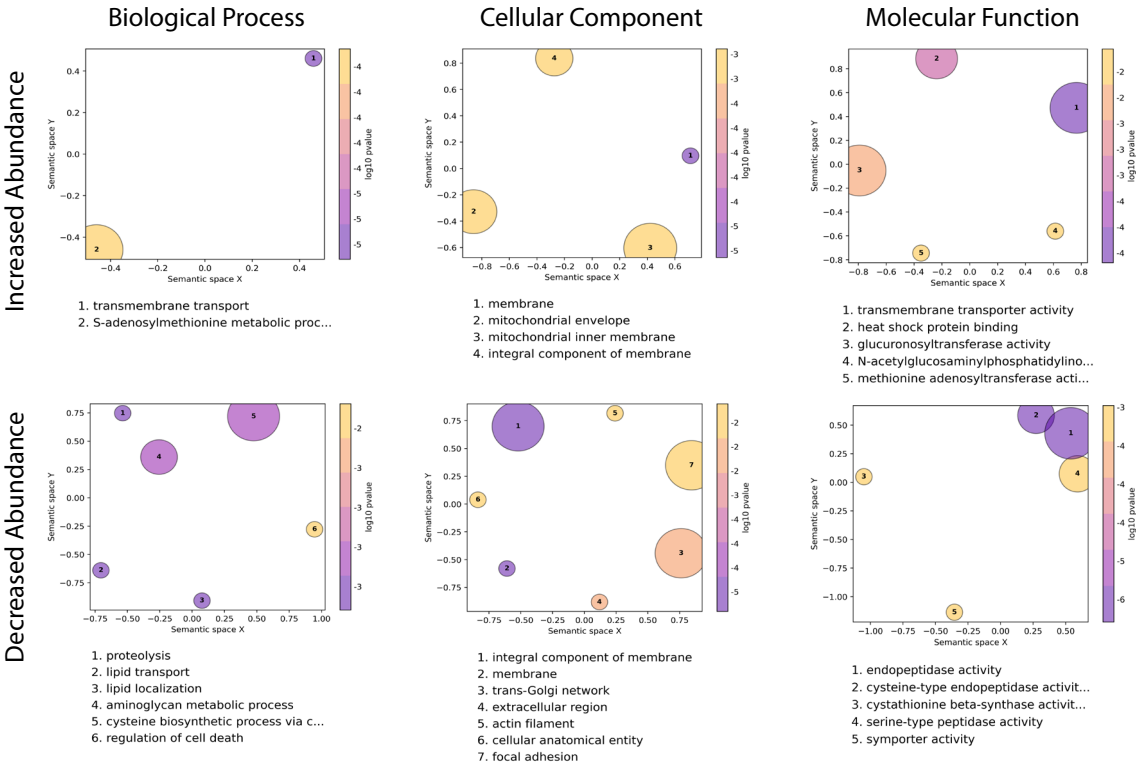

B.

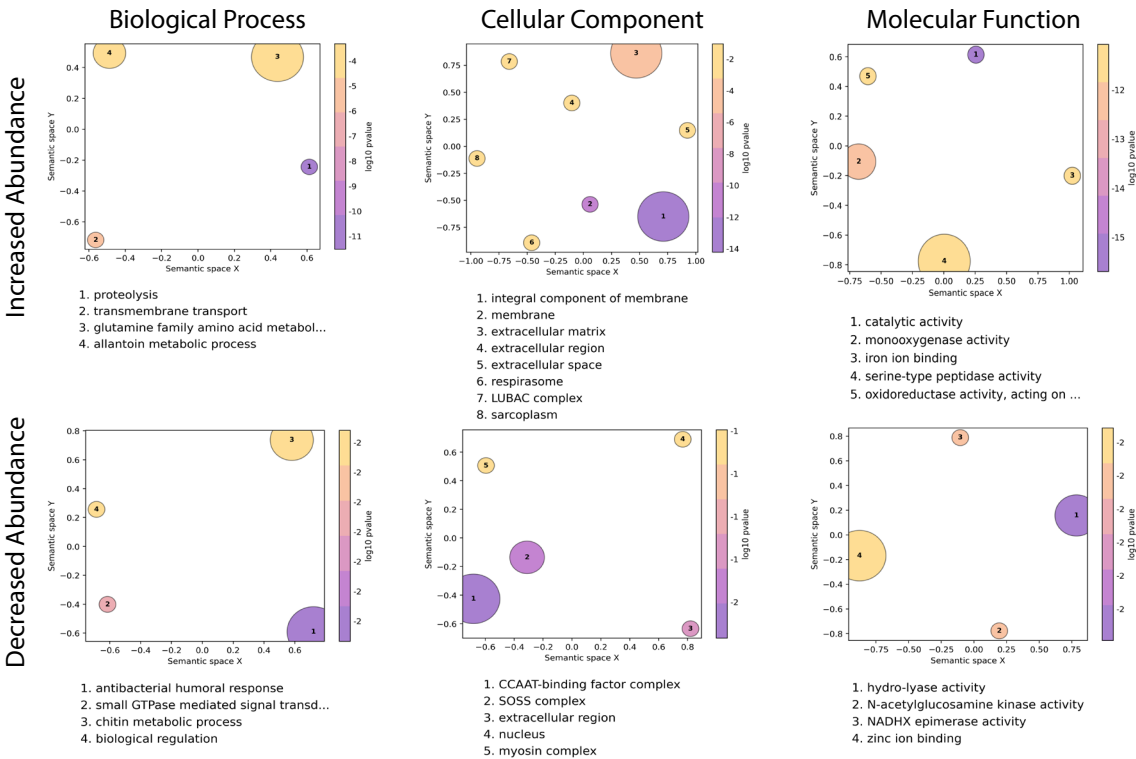

C.

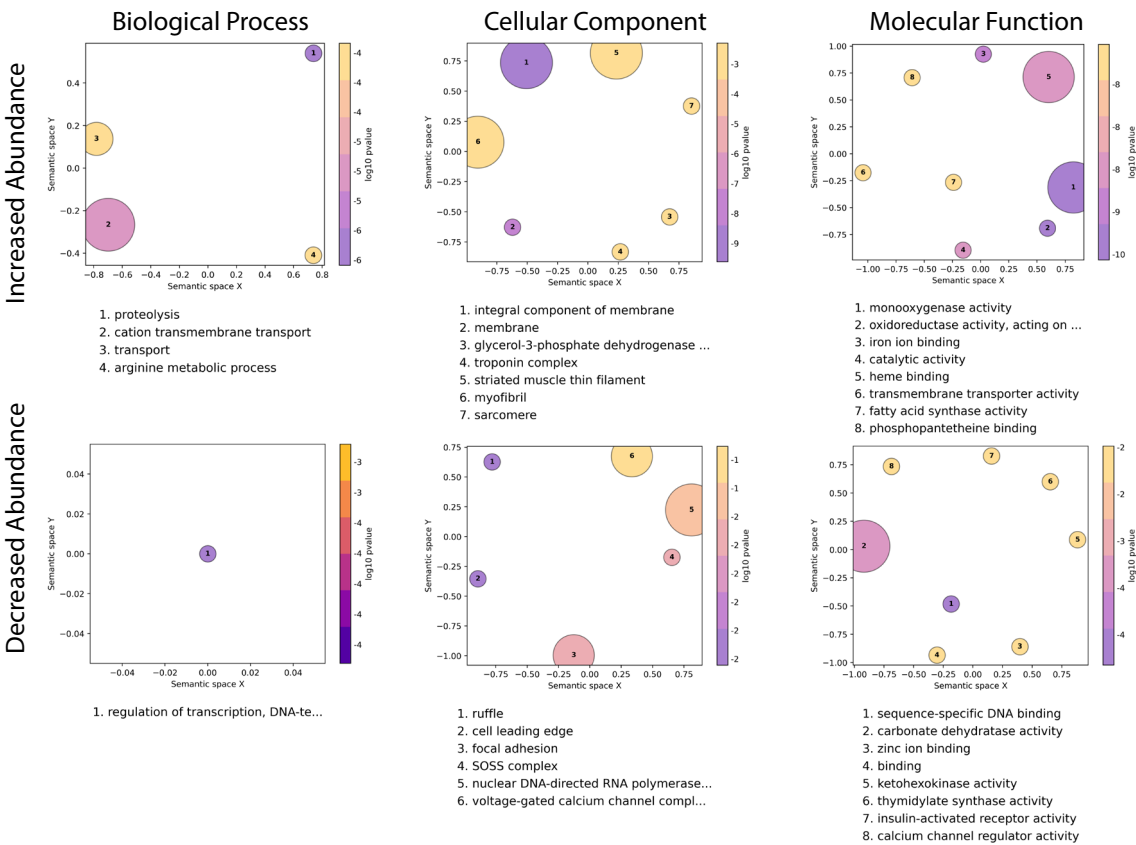

Supplement: S2 Fig — GO terms associated with the differentially expressed transcripts in COL.wMel midguts at 7dpf (A) and carcasses at 4 and 7dpf (B,C). The top 10 GO terms from each category (Biological Process, Cellular Component, Molecular Function), determined by topGO, were run in the GO Figure! pipeline to combine semantically similar terms and reduce redundancy. Terms are ranked by lowest log10(p-value). The size of each graphical point corresponds to the number of topGO terms associated with the listed summarizing term. (PDF) [file pntd.0011674.s002.pdf]

A.

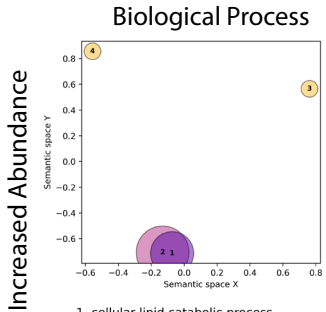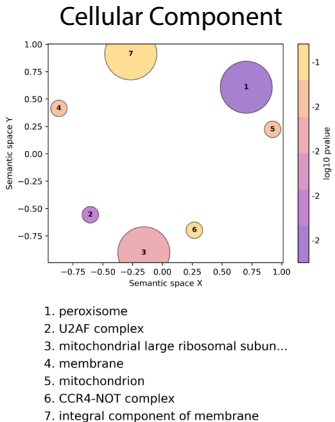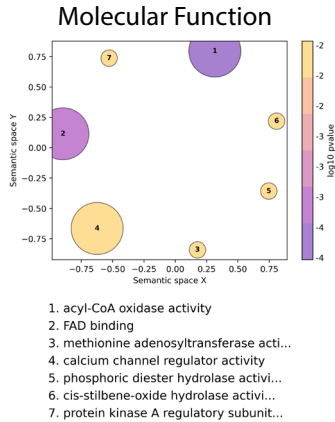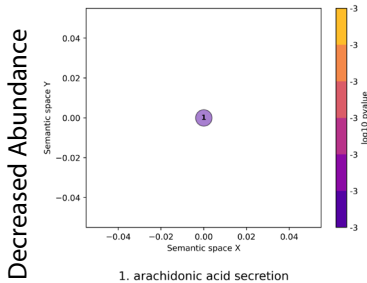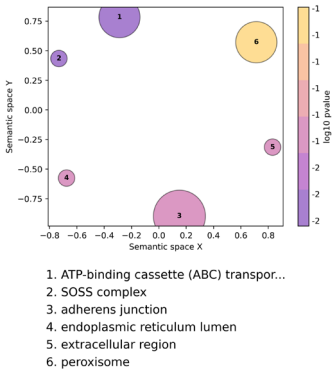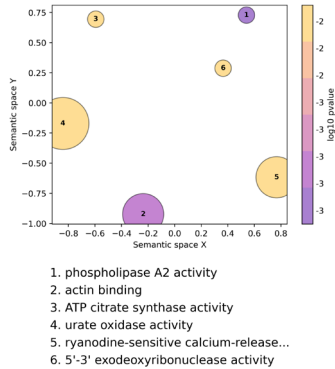

B.

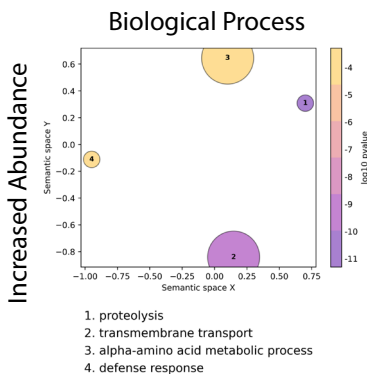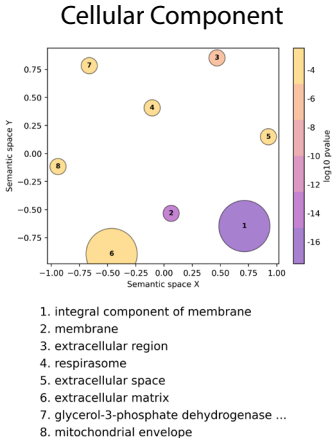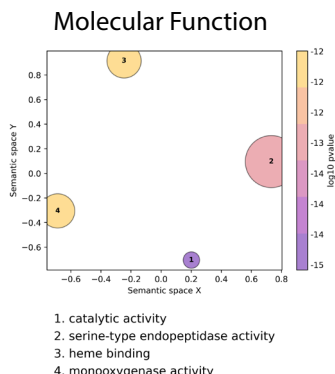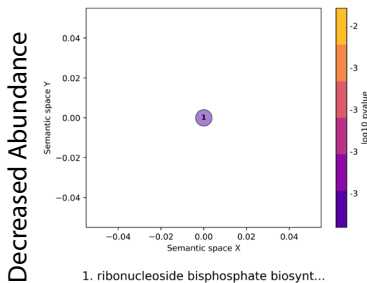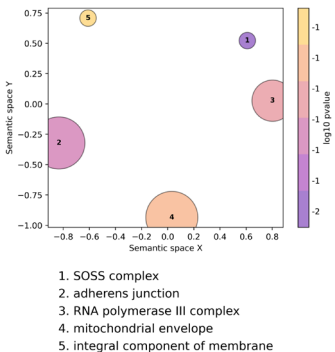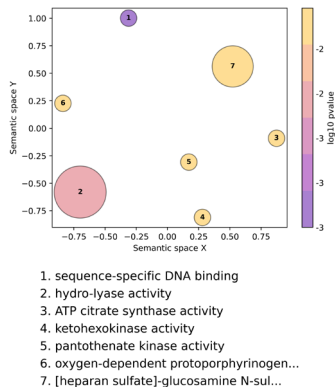

C.

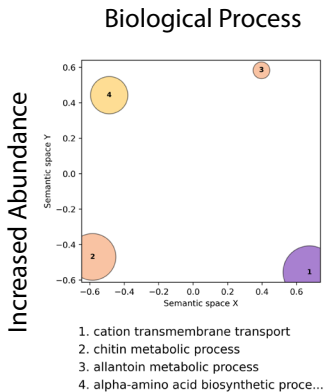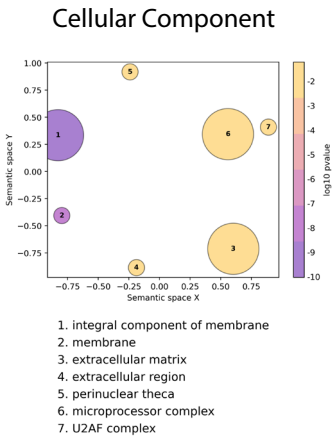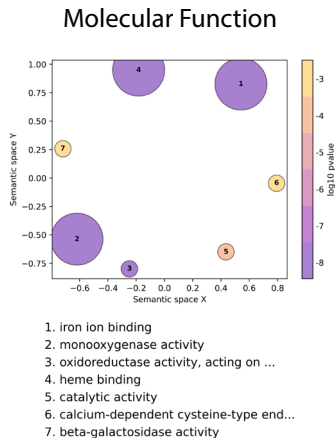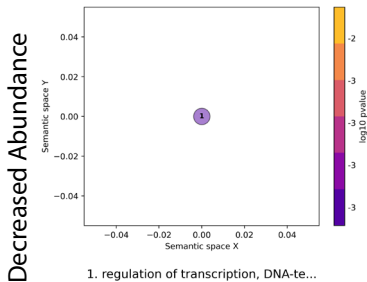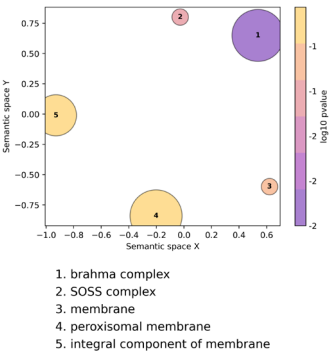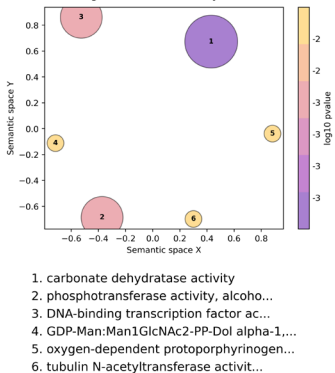

Supplement: S3 Fig — GO terms associated with the differentially expressed transcripts in COL.wMel midguts at 7dpf (A) and carcasses at 4 and 7dpf (B,C) on a ZIKV-infected bloodmeal. The top 10 GO terms from each category (Biological Process, Cellular Component, Molecular Function), determined by topGO, were run in the GO Figure! pipeline to combine semantically similar terms and reduce redundancy. Terms are ranked by lowest log10(p-value). The size of each graphical point corresponds to the number of topGO terms associated with the listed summarizing term. (PDF) [file pntd.0011674.s003.pdf]

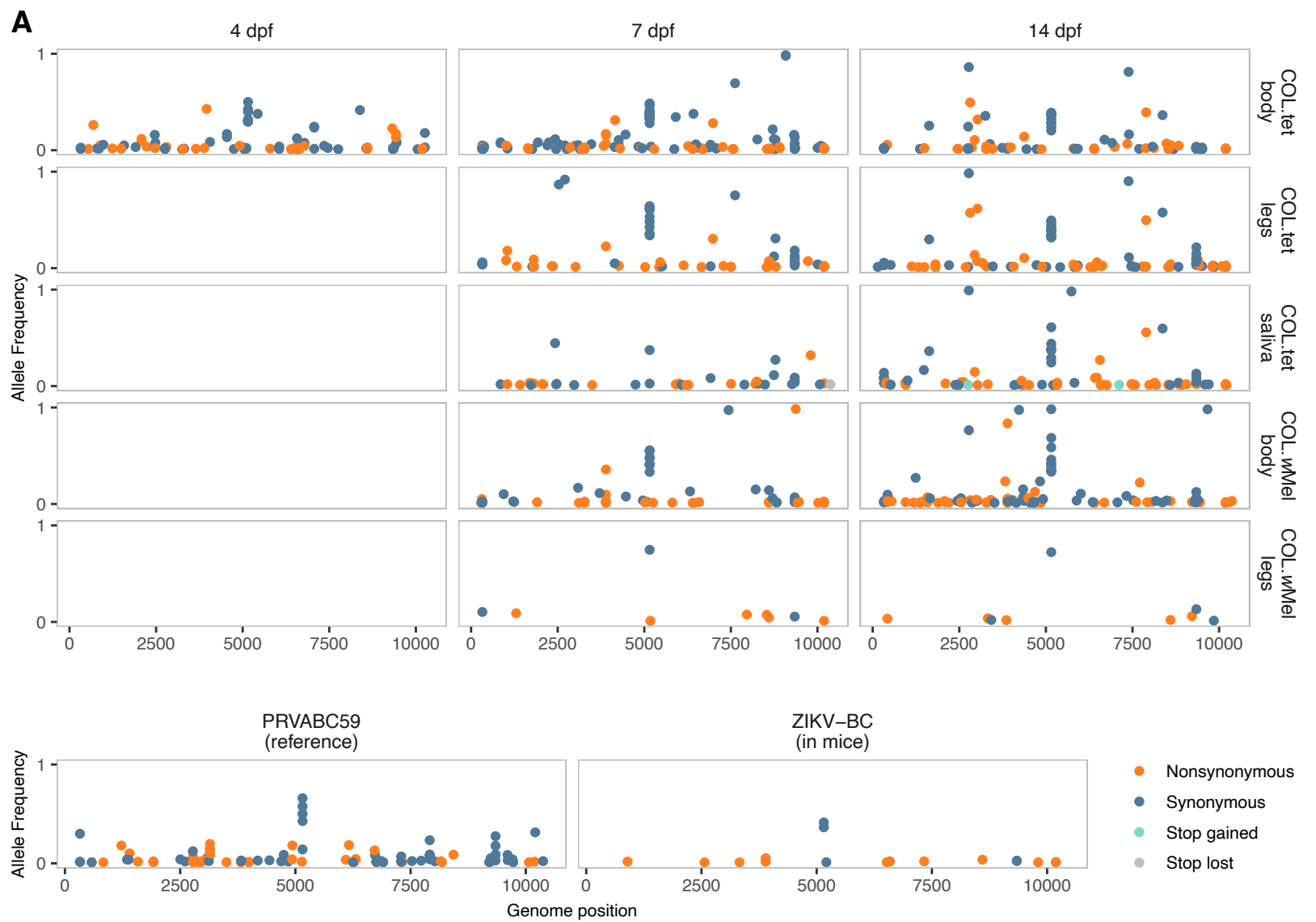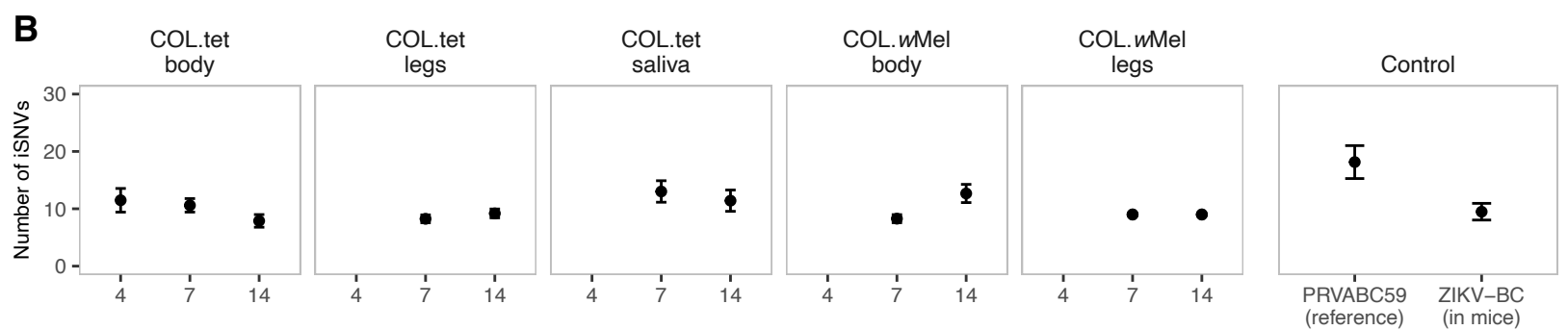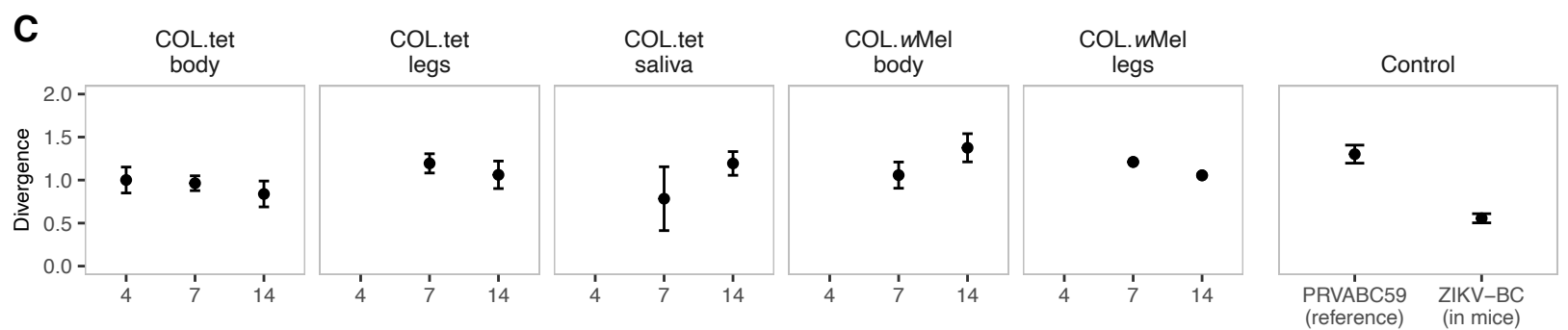

Supplement: S4 Fig — (A). iSNVs ≥ 1% are plotted along the PRVABC59 genome and colored by mutation type: nonsynonymous (orange), synonymous (blue), stop gained (green), and stop lost (grey). (B). Number of iSNVs per sample are plotted across groups and time points. (C). The per-sample divergence (total allele frequency) is plotted as in B. All groups in B and C underwent 10,000 Bayesian bootstrap replicates, from which mean values and standard deviations were calculated and plotted. (PDF) [file pntd.0011674.s004.pdf]

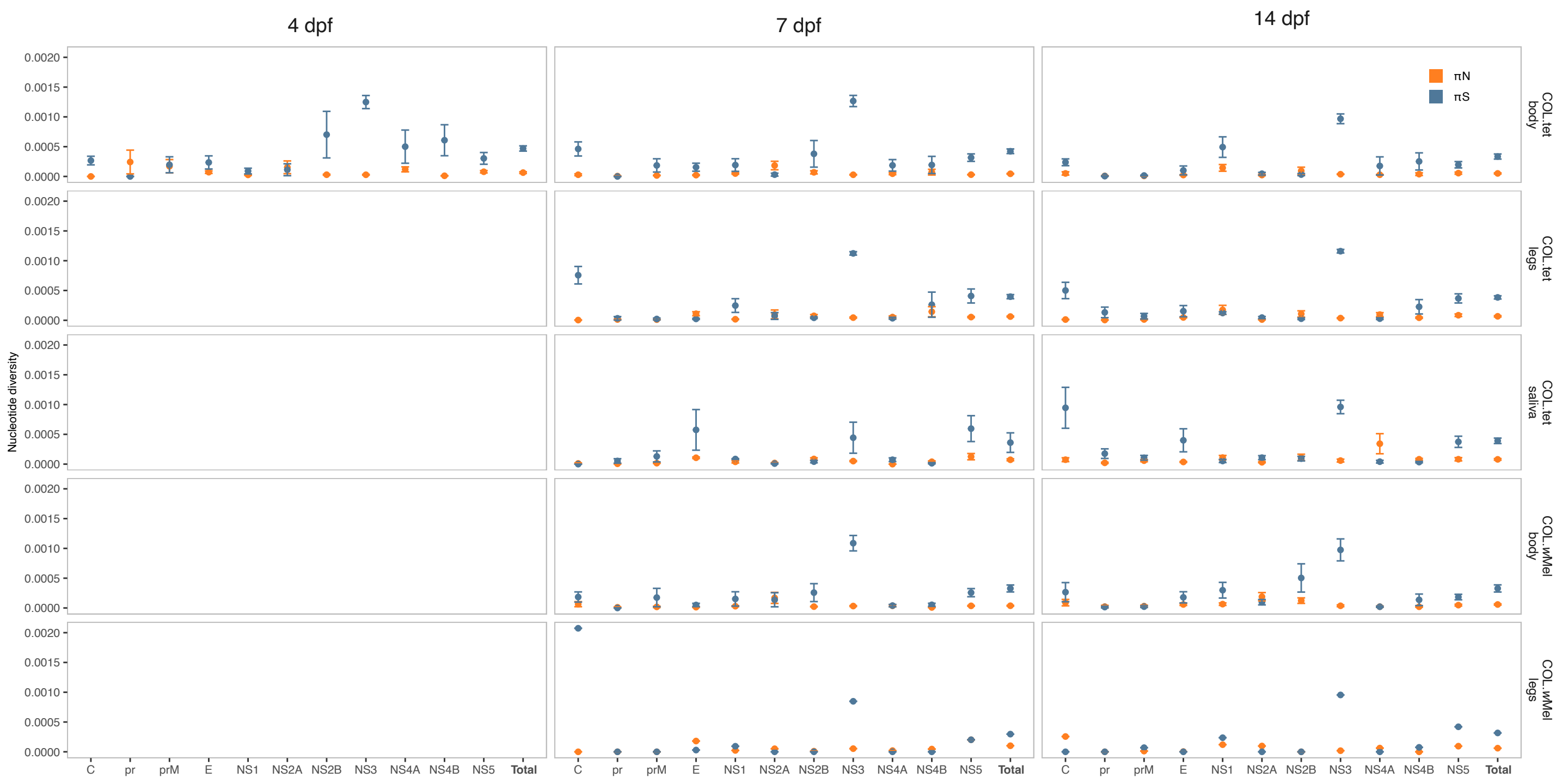

Supplement: S6 Fig — Per-gene nucleotide diversity is quantified for nonsynonymous (πN; orange) and synonymous (πS; blue) sites across all ZIKV plaque-positive tissues collected from COL.wMel and COL.tet mosquitoes. All groups underwent 10,000 Bayesian bootstrap replicates, from which mean values and standard deviations were calculated and plotted. (PDF) [file pntd.0011674.s006.pdf]

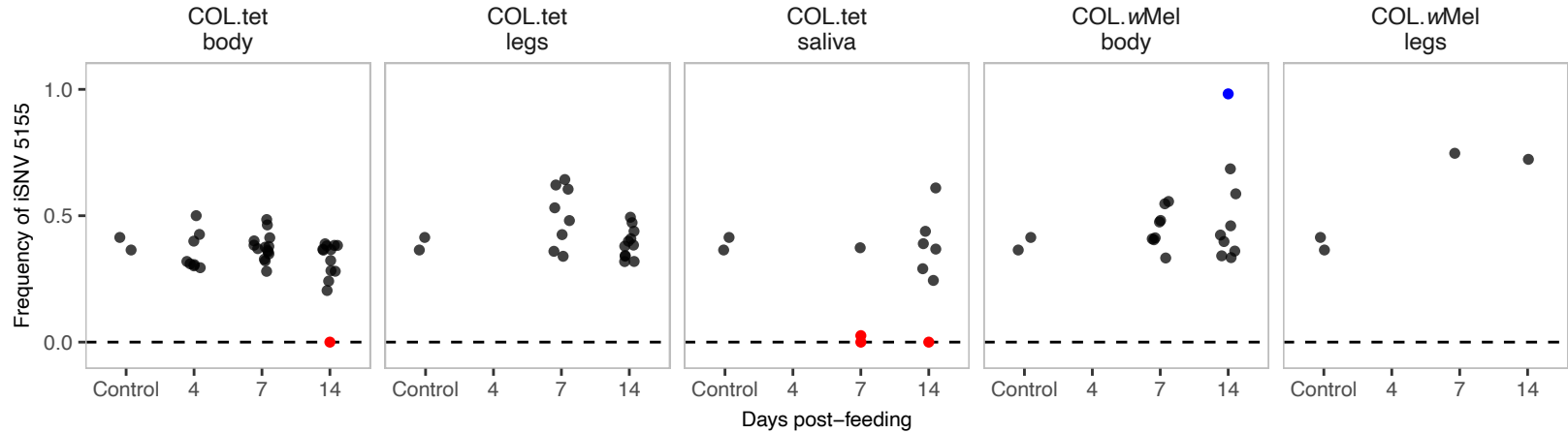

Supplement: S7 Fig — The allele frequency of iSNV 5155 is plotted over time in all experimental groups. Samples were colored by allele frequencies: <5% (red), 5–95% (black), >95% (blue). If iSNV 5155 was not detected in a sample, it was assigned the allele frequency 0. (PDF) [file pntd.0011674.s007.pdf]
