# Supplementary material for: Wolbachia-mediated resistance to Zika virus infection in Aedes aegypti is dominated by diverse transcriptional regulation and weak evolutionary pressures
Source: PLoS Negl Trop Dis. 2023 Oct 2;17(10):e0011674. doi: 10.1371/journal.pntd.0011674 (PMC10569609; doi:10.1371/journal.pntd.0011674)
Supplement: S5 Fig — The proportion of variants per mutation type was calculated for nonsynonymous (orange) and synonymous (blue) mutation types. For each group and mutation type, the number of mutations that fell within a within-host iSNV frequency bin was divided by the total number of mutations (≤ 50% allele frequency). The grey dots and connecting lines denote the neutral expectation proportion for each frequency bin, assuming neutral selection and constant population size, modeled as following an inverse distribution. (PDF) [file pntd.0011674.s005.pdf]

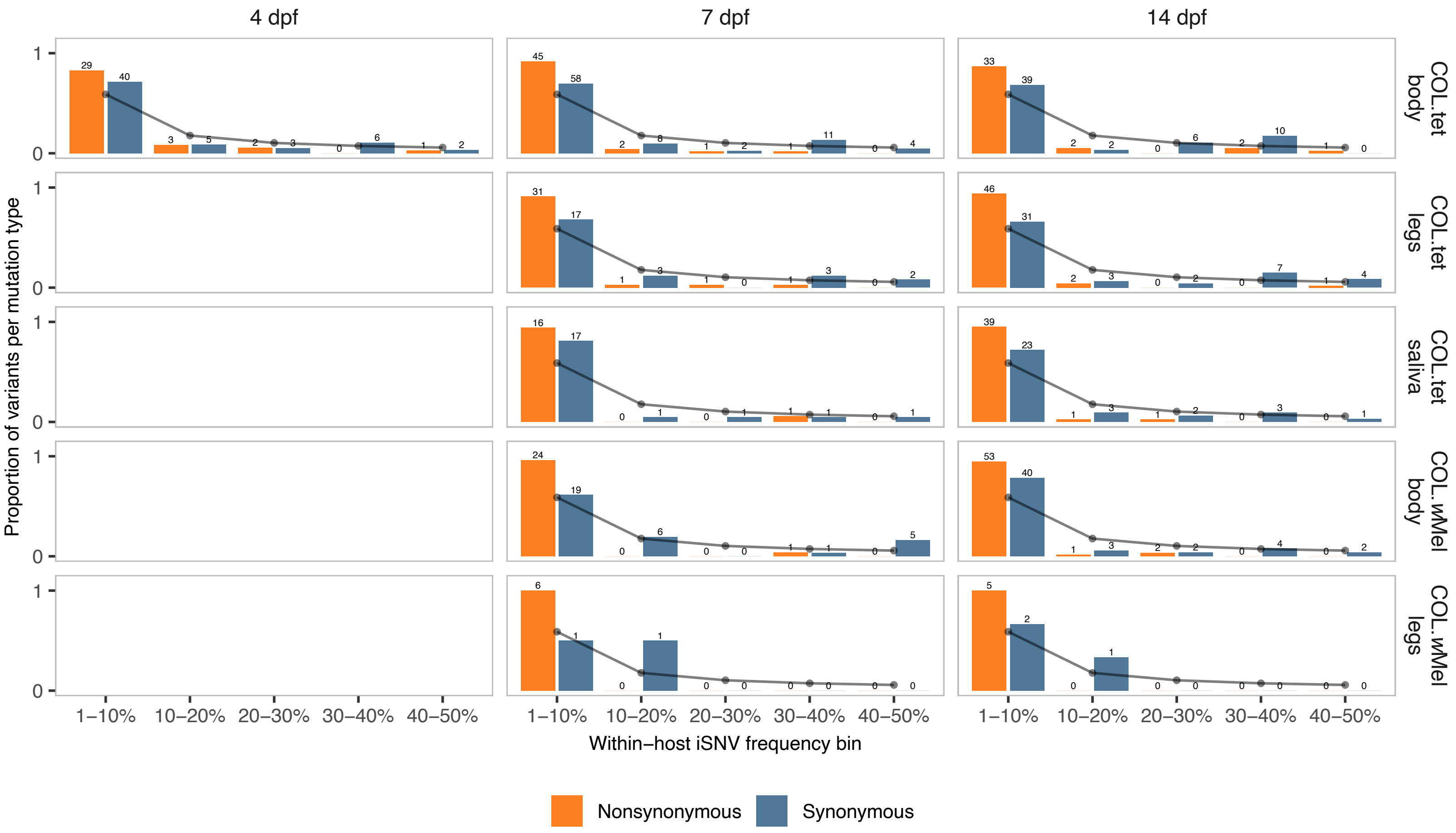

4 dpf
7 dpf
14 dpf
COL.tet body
COL.tet legs
COL.tet saliva
COL.wMel body
COL.wMel legs
Proportion of variants per mutation type
0
1
1–10%
10–20%
20–30%
30–40%
40–50%
Within–host iSNV frequency bin
Nonsynonymous
Synonymous
