# Supplementary material for: Wolbachia-mediated resistance to Zika virus infection in Aedes aegypti is dominated by diverse transcriptional regulation and weak evolutionary pressures
Source: PLoS Negl Trop Dis. 2023 Oct 2;17(10):e0011674. doi: 10.1371/journal.pntd.0011674 (PMC10569609; doi:10.1371/journal.pntd.0011674)
Supplement: S1 Table — (PDF) [file pntd.0011674.s008.pdf]

S1 Table. Mosquito innate immune genes differentially expressed in COL.wMel relative to COL.tet.						
**Vectorbase ID**	**Gene Name**	**Product Description**	**GO term: CC**	**GO term: MF**	**GO term: BP**	**Sample Group**
AAEL000037	CLIPB35	Clip-Domain Serine Protease family B.	extracellular region	hydrolase activity;peptidase activity;serine-type endopeptidase activity;serine-type peptidase activity	proteolysis	carcass_4, carcass_7
AAEL000227	SCRB8	Class B Scavenger Receptor (CD36 domain).	integral component of membrane;membrane	N/A	N/A	carcass_4
AAEL000234	SCRB7	Class B Scavenger Receptor (CD36 domain).	membrane	N/A	N/A	carcass_4
AAEL000652	GNBPA2	Gram-Negative Binding Protein (GNBP) or Beta-1 3-Glucan Binding Protein (BGBP).	N/A	carbohydrate binding;hydrolase activity, hydrolyzing O-glycosyl compounds	carbohydrate metabolic process	midgut_7
AAEL000726	N/A	fibrinogen and fibronectin	N/A	N/A	N/A	carcass_7
AAEL000749	N/A	Angiopoietin-like protein variant (Fragment) [Source:UniProtKB/TrEMBL;Acc:Q1HRV2]	N/A	N/A	N/A	carcass_7
AAEL000760	CLIPB30	Clip-Domain Serine Protease family B.	extracellular region	hydrolase activity;peptidase activity;serine-type endopeptidase activity;serine-type peptidase activity	proteolysis	carcass_7
AAEL001401	LRIM10A	leucine-rich immune protein (Short)	N/A	protein binding	N/A	carcass_4, carcass_7
AAEL001402	LRIM10B	leucine-rich immune protein (Short)	N/A	protein binding	N/A	carcass_4, carcass_7
AAEL001414	LRIM9	leucine-rich immune protein (Short)	N/A	protein binding	N/A	carcass_4, carcass_4, carcass_7
AAEL001417	LRIM7	leucine-rich immune protein (Short)	N/A	protein binding	N/A	carcass_4, carcass_7
AAEL001420	LRIM8	leucine-rich immune protein (Short)	N/A	protein binding	N/A	carcass_4, carcass_7
AAEL001650	N/A	ML domain-containing protein [Source:UniProtKB/TrEMBL;Acc:A0A1S4EZF1]	N/A	N/A	N/A	midgut_7
AAEL001794	N/A	macroglobulin/complement	extracellular region;extracellular space	endopeptidase inhibitor activity	negative regulation of endopeptidase activity	carcass_4, carcass_7
AAEL002309	TPX4	Thioredoxin Peroxidase.	obsolete cell	antioxidant activity;oxidoreductas	cell redox homeostasis;cellular	midgut_7

				e activity;peroxiredoxin activity	oxidant detoxification;obsolete oxidation-reduction process	
AAEL002601	CLIPA1	Clip-Domain Serine Protease family A. Protease homologue.	N/A	serine-type endopeptidase activity	proteolysis	carcass_4
AAEL002720	SRPN20	Serine Protease Inhibitor (serpin) likely cleavage at V/V.	extracellular space	N/A	N/A	carcass_4
AAEL002731	SRPN14	Serine Protease Inhibitor (serpin) homologue - unlikely to be inhibitory.	extracellular space	N/A	N/A	carcass_4, carcass_7
AAEL003156	N/A	fibrinogen and fibronectin	N/A	N/A	N/A	carcass_4
AAEL003182	SRPN26	Serine Protease Inhibitor (serpin) homologue - unlikely to be inhibitory.	extracellular space	N/A	N/A	midgut_4
AAEL003253	CLIPB13B	Clip-Domain Serine Protease family B.	N/A	serine-type endopeptidase activity	proteolysis	carcass_4, carcass_7
AAEL003294	N/A	fibrinogen and fibronectin	N/A	N/A	N/A	midgut_7
AAEL003389	ATT	attacin anti-microbial peptide	extracellular region;extracellular space	N/A	antibacterial humoral response;defense response to bacterium	carcass_4
AAEL003439	CASPS18	caspase (short)	N/A	cysteine-type endopeptidase activity;cysteine-type peptidase activity	proteolysis	midgut_7
AAEL003444	CASPS19	caspase (short)	N/A	cysteine-type endopeptidase activity;cysteine-type peptidase activity;hydrolase activity;peptidase activity	proteolysis	midgut_7
AAEL003631	CLIPB41	Clip-Domain Serine Protease family B.	N/A	hydrolase activity;peptidase activity;serine-type endopeptidase activity;serine-type peptidase activity	proteolysis	carcass_4
AAEL003697	SRPN17	Serine Protease Inhibitor (serpin) homologue - unlikely to be inhibitory.	extracellular space	N/A	N/A	carcass_4, carcass_7
AAEL003712	LYSC10	C-Type Lysozyme (Lys-E).	N/A	lysozyme activity	N/A	midgut_7
AAEL003723	LYSC11	C-Type Lysozyme (Lys-A).	N/A	lysozyme activity	N/A	midgut_4
AAEL004390	HPX8B	heme peroxidase	N/A	heme binding;peroxidase activity	cellular oxidant detoxification;obsolete oxidation-reduction process;oogenesis;response to oxidative stress	midgut_4

AAEL004833	N/A	unspecified product	extracellular region	N/A	defense response to bacterium	carcass_4, carcass_4
AAEL004978	N/A	DEAD box ATP-dependent RNA helicase	N/A	ATP binding;helicase activity;hydrolase activity;nucleic acid binding;nucleotide binding	N/A	midgut_4
AAEL004979	CLIPD2	Clip-Domain Serine Protease family D.	N/A	hydrolase activity;peptidase activity;serine-type endopeptidase activity;serine-type peptidase activity	proteolysis	carcass_4
AAEL005108	MNSOD2	manganese-iron (Mn-Fe) superoxide dismutase	N/A	metal ion binding;oxidoreductase activity;superoxide dismutase activity	obsolete oxidation-reduction process;removal of superoxide radicals;superoxide metabolic process	carcass_4, carcass_7
AAEL005293	GALE8A	Galectin [Source:UniProtKB/TrEMBL;Acc:Q16ND5]	N/A	carbohydrate binding	N/A	midgut_7
AAEL005482	CTL18	C-Type Lectin (CTL).	N/A	carbohydrate binding	N/A	midgut_4
AAEL005641	CTLGA5	C-Type Lectin (CTL) - galactose binding.	N/A	N/A	N/A	midgut_7
AAEL005956	CASPS16	caspase (short)	N/A	cysteine-type endopeptidase activity;cysteine-type peptidase activity	proteolysis	midgut_4, carcass_4
AAEL006161	CLIPB31	Clip-Domain Serine Protease family B	extracellular region	hydrolase activity;peptidase activity;serine-type endopeptidase activity;serine-type peptidase activity	proteolysis	carcass_4
AAEL006355	SCRC1	Class C Scavenger Receptor (Sushi/SCR/CCP MAM and Somatomedin B domains).	integral component of membrane;membrane	N/A	N/A	midgut_7
AAEL006361	SCRC2	Class C Scavenger Receptor (Sushi/SCR/CCP MAM and Somatomedin B domains).	integral component of membrane;membrane	N/A	N/A	midgut_4, midgut_7
AAEL006377	LRIM31	leucine-rich immune protein (Coil-less)	N/A	protein binding	N/A	carcass_4
AAEL006674	CLIPB29	Clip-Domain Serine Protease family B.	extracellular region	hydrolase activity;peptidase activity;serine-type endopeptidase activity;serine-type peptidase activity	proteolysis	carcass_4
AAEL006704	N/A	fibrinogen and fibronectin	N/A	N/A	N/A	midgut_7
AAEL006854	N/A	Niemann-Pick Type C-2, putative	N/A	N/A	intracellular cholesterol transport	midgut_4, midgut_7
AAEL007103	LRIM15	leucine-rich immune protein (TM)	integral component of	protein binding	N/A	carcass_4

			membrane;membrane			
AAEL007224	LRIM22	leucine-rich immune protein (Coil-less)	N/A	protein binding	N/A	carcass_4, carcass_7
AAEL007624	REL2	IMD pathway signalling NF-kappaB Relish-like transcription factor	cytoplasm;host cell nucleus;nucleus	DNA binding;DNA-binding transcription factor activity;protein binding	regulation of transcription, DNA-templated	midgut_7
AAEL007696	REL1A	TOLL pathway signalling NF-kappaB Relish-like transcription factor	cytoplasm;host cell nucleus;nucleus	DNA binding;DNA-binding transcription factor activity	regulation of transcription, DNA-templated	carcass_4, carcass_7
AAEL007823	N/A	PIWI	N/A	nucleic acid binding;protein binding	gene silencing by RNA	midgut_7
AAEL007993	CLIPB27	Clip-Domain Serine Protease family B.	extracellular region	hydrolase activity;peptidase activity;serine-type endopeptidase activity;serine-type peptidase activity	proteolysis	midgut_4, midgut_7
AAEL008098	PIWI2	PIWI	N/A	nucleic acid binding;protein binding	gene silencing by RNA	midgut_4
AAEL008370	SCRB17	Class B Scavenger Receptor (CD36 domain).	integral component of membrane;membrane	N/A	N/A	carcass_4, carcass_7
AAEL008658	LRIM16	leucine-rich immune protein (TM)	integral component of membrane;membrane	protein binding	N/A	midgut_4, midgut_7
AAEL008668	CLIPB22	Clip-Domain Serine Protease family B.	N/A	serine-type endopeptidase activity	proteolysis	carcass_4
AAEL009178	GNBPB4	Gram-Negative Binding Protein (GNBP) or Beta-1 3-Glucan Binding Protein (BGBP).	N/A	hydrolase activity, hydrolyzing O-glycosyl compounds	carbohydrate metabolic process	carcass_7
AAEL009384	N/A	fibrinogen and fibronectin	N/A	N/A	N/A	midgut_7
AAEL009474	PGRPS1	Peptidoglycan Recognition Protein (Short)	N/A	N-acetylmuramoyl-L-alanine amidase activity;peptidoglycan binding;zinc ion binding	immune system process;innate immune response;peptidoglycan catabolic process	carcass_4
AAEL009556	N/A	Niemann-Pick Type C-2, putative	N/A	N/A	intracellular cholesterol transport	midgut_4, midgut_7
AAEL009792	LRIM25	leucine-rich immune protein (Coil-less)	N/A	protein binding	N/A	carcass_7
AAEL009842	GALE12	Galectin [Source:UniProtKB/TrEMBL;Acc:Q16UP1]	N/A	N/A	N/A	midgut_4, midgut_7
AAEL009850	GALE14	Galectin [Source:UniProtKB/TrEMBL;Acc:Q16UP0]	N/A	carbohydrate binding	N/A	midgut_4, midgut_7

AAEL009894	LRIM21	leucine-rich immune protein (Coil-less)	integral component of membrane;membrane	protein binding	N/A	midgut_4, midgut_7
AAEL010125	LRIM17	leucine-rich immune protein (Coil-less)	N/A	protein binding	N/A	carcass_4
AAEL010128	LRIM4	leucine-rich immune protein (Long)	N/A	protein binding	N/A	carcass_4, carcass_7
AAEL010132	LRIM3	leucine-rich immune protein (Long)	N/A	protein binding	N/A	carcass_4
AAEL010769	SRPN6	Serine Protease Inhibitor (serpin) likely cleavage at S/A.	extracellular space	N/A	N/A	carcass_7
AAEL011009	N/A	fibrinogen and fibronectin	N/A	N/A	N/A	carcass_4, carcass_7
AAEL011407	CTL20	C-Type Lectin (CTL20)	N/A	N/A	N/A	midgut_4
AAEL011408	CTL21	C-Type Lectin (CTL21)	N/A	N/A	N/A	carcass_4, carcass_7
AAEL011453	CTL14	C-Type Lectin (CTL14)	N/A	N/A	N/A	carcass_4, carcass_7
AAEL011455	CTLMA12	C-Type Lectin (CTLMA12) - mannose binding	N/A	carbohydrate binding	N/A	carcass_4
AAEL011621	CTLMA13	C-Type Lectin (CTL) - mannose binding.	N/A	carbohydrate binding;serine-type endopeptidase activity	proteolysis	carcass_4, carcass_7
AAEL011633	N/A	fibrinogen and fibronectin	N/A	N/A	N/A	carcass_4
AAEL011763	PPO3	prophenoloxidase	N/A	metal ion binding;oxidoreductase activity	obsolete oxidation-reduction process	carcass_4
AAEL011764	PPO10	prophenoloxidase	N/A	metal ion binding;oxidoreductase activity	obsolete oxidation-reduction process	carcass_4, carcass_7
AAEL012003	GALE6B	Galectin [Source:UniProtKB/TrEMBL;Acc:Q17AG2]	N/A	carbohydrate binding	N/A	midgut_7
AAEL012086	LRIM1	leucine-rich immune protein (Long)	N/A	protein binding	N/A	carcass_4, carcass_7
AAEL012255	LRIM13	leucine-rich immune protein (Short)	N/A	protein binding	N/A	carcass_4, carcass_7
AAEL012380	PGRPLA	Peptidoglycan Recognition Protein (Long)	integral component of membrane;membrane	N-acetylmuramoyl-L-alanine amidase activity;zinc ion binding	peptidoglycan catabolic process	midgut_4, midgut_7
AAEL012471	DOME	JAKSTAT pathway signalling Transmembrane Receptor Domeless.	integral component of membrane;membrane	cytokine receptor activity;protein binding	cytokine-mediated signaling pathway	midgut_7
AAEL012763	LRIM24	leucine-rich immune protein (Coil-less)	N/A	protein binding	N/A	carcass_4

AAEL012767	LRIM5	leucine-rich immune protein (Short)	N/A	protein binding	N/A	carcass_4
AAEL012911	LRIM18	leucine-rich immune protein (Coil-less)	integral component of membrane;membran e	protein binding	N/A	carcass_4
AAEL013245	CLIPB28	Clip-Domain Serine Protease family B.	N/A	peptidase activity;serine-type endopeptidase activity	proteolysis	carcass_7, carcass_7
AAEL013417	N/A	fibrinogen and fibronectin	N/A	N/A	N/A	carcass_4
AAEL013496	PPO8	prophenoloxidase	N/A	metal ion binding;oxidoreducta se activity	obsolete oxidation-reduction process	carcass_4
AAEL013692	PIWI3	PIWI	N/A	nucleic acid binding;protein binding	gene silencing by RNA	midgut_4
AAEL014348	CASPS8	caspase (short)	N/A	cysteine-type endopeptidase activity;cysteine-type peptidase activity;hydrolase activity;peptidase activity	proteolysis	midgut_7, midgut_7
AAEL014382	CTLMA14	C-Type Lectin (CTL) - mannose binding.	N/A	carbohydrate binding	N/A	carcass_4
AAEL014544	PPO6	prophenoloxidase	N/A	metal ion binding;oxidoreducta se activity	obsolete oxidation-reduction process	carcass_4, carcass_7
AAEL017536	GRRP	holotricin glycine rich repeat protein (GRRP) anti-microbial peptide	N/A	N/A	N/A	carcass_4, carcass_4, carcass_4, carcass_7, carcass_7

CC, cellular component; MF, molecular function; BP, biological process.
