# Supplementary material for: Wolbachia-mediated resistance to Zika virus infection in Aedes aegypti is dominated by diverse transcriptional regulation and weak evolutionary pressures
Source: PLoS Negl Trop Dis. 2023 Oct 2;17(10):e0011674. doi: 10.1371/journal.pntd.0011674 (PMC10569609; doi:10.1371/journal.pntd.0011674)
Supplement: S2 Table — (PDF) [file pntd.0011674.s009.pdf]

S2 Table. Mosquito innate immune genes differentially expressed in COL.wMel relative to COL.tet during ZIKV infection.

Vectorbase ID	Gene Name	Product Description	GO term: CC	GO term: MF	GO term: BP	Sample Group
AAEL000037	CLIPB35	Clip-Domain Serine Protease family B.	extracellular region	hydrolase activity;peptidase activity;serine-type endopeptidase activity;serine-type peptidase activity	proteolysis	carcass_4
AAEL000057	TOLL5B	Toll-like receptor	integral component of membrane;membrane	protein binding	immune system process;innate immune response;signal transduction	carcass_4
AAEL000227	SCRB8	Class B Scavenger Receptor (CD36 domain).	integral component of membrane;membrane	N/A	N/A	carcass_4
AAEL000234	SCRB7	Class B Scavenger Receptor (CD36 domain).	membrane	N/A	N/A	carcass_7
AAEL000652	GNBPA2	Gram-Negative Binding Protein (GNBP) or Beta-1 3-Glucan Binding Protein (BGBP).	N/A	carbohydrate binding;hydrolase activity, hydrolyzing O-glycosyl compounds	carbohydrate metabolic process	midgut_4
AAEL000760	CLIPB30	Clip-Domain Serine Protease family B.	extracellular region	hydrolase activity;peptidase activity;serine-type endopeptidase activity;serine-type peptidase activity	proteolysis	carcass_4
AAEL001077	CLIPB45	Clip-Domain Serine Protease family B. Protease homologue.	N/A	serine-type endopeptidase activity	proteolysis	carcass_4, carcass_7
AAEL001401	LRIM10A	leucine-rich immune protein (Short)	N/A	protein binding	N/A	carcass_4
AAEL001402	LRIM10B	leucine-rich immune protein (Short)	N/A	protein binding	N/A	carcass_4
AAEL001414	LRIM9	leucine-rich immune protein (Short)	N/A	protein binding	N/A	carcass_4, carcass_4, carcass_7, carcass_7
AAEL001417	LRIM7	leucine-rich immune protein (Short)	N/A	protein binding	N/A	carcass_4
AAEL001420	LRIM8	leucine-rich immune protein (Short)	N/A	protein binding	N/A	carcass_4, carcass_7
AAEL001794	N/A	macroglobulin/complement	extracellular region;extracellular space	endopeptidase inhibitor activity	negative regulation of endopeptidase activity	carcass_4
AAEL002126	CLIPA15	Clip-Domain Serine Protease family A. Protease homologue.	N/A	serine-type endopeptidase activity	proteolysis	carcass_4
AAEL002601	CLIPA1	Clip-Domain Serine Protease family A.	N/A	serine-type endopeptidase	proteolysis	carcass_4

		Protease homologue.		activity		
AAEL002720	SRPN20	Serine Protease Inhibitor (serpin) likely cleavage at V/V.	extracellular space	N/A	N/A	carcass_4
AAEL002731	SRPN14	Serine Protease Inhibitor (serpin) homologue - unlikely to be inhibitory.	extracellular space	N/A	N/A	carcass_4
AAEL003253	CLIPB13B	Clip-Domain Serine Protease family B.	N/A	serine-type endopeptidase activity	proteolysis	carcass_4
AAEL003631	CLIPB41	Clip-Domain Serine Protease family B.	N/A	hydrolase activity;peptidase activity;serine-type endopeptidase activity;serine-type peptidase activity	proteolysis	carcass_4, carcass_7
AAEL003697	SRPN17	Serine Protease Inhibitor (serpin) homologue - unlikely to be inhibitory.	extracellular space	N/A	N/A	carcass_4
AAEL003832	DEFC	defensin anti-microbial peptide	extracellular region	N/A	defense response;defense response to bacterium;immune system process;innate immune response	carcass_4
AAEL003841	DEFA	Defensin-A [Source:UniProtKB/Swiss-Prot;Acc:P91793]	N/A	N/A	defense response	carcass_4
AAEL003857	N/A	INVERT_DEFENSINS domain-containing protein [Source:UniProtKB/TrEMBL;Acc:A0A1S4F676]	N/A	N/A	defense response	carcass_4
AAEL004120	N/A	Niemann-Pick Type C-2, putative	N/A	N/A	intracellular cholesterol transport	carcass_4
AAEL004524	CLIPC5B	Clip-Domain Serine Protease family C.	N/A	hydrolase activity;peptidase activity;serine-type endopeptidase activity;serine-type peptidase activity	proteolysis	carcass_4
AAEL004833	N/A	unspecified product	extracellular region	N/A	defense response to bacterium	carcass_4
AAEL004979	CLIPD2	Clip-Domain Serine Protease family D.	N/A	hydrolase activity;peptidase activity;serine-type endopeptidase activity;serine-type peptidase activity	proteolysis	carcass_7
AAEL005093	CLIPB46	Clip-Domain Serine Protease family B.	extracellular region	hydrolase activity;peptidase activity;serine-type endopeptidase activity;serine-type peptidase activity	proteolysis	carcass_4
AAEL005108	MNSOD2	manganese-iron (Mn-Fe) superoxide dismutase	N/A	metal ion binding;oxidoreductase activity;superoxide	obsolete oxidation-reduction process;removal of	carcass_4, carcass_7

				dismutase activity	superoxide radicals;superoxide metabolic process	
AAEL005431	CLIPB37	Clip-Domain Serine Protease family B.	N/A	hydrolase activity;peptidase activity;serine-type endopeptidase activity;serine-type peptidase activity	proteolysis	carcass_4
AAEL005792	CLIPE8	Clip-Domain Serine Protease family E. Protease homologue.	N/A	peptidase activity;serine-type endopeptidase activity	proteolysis	carcass_4
AAEL006161	CLIPB31	Clip-Domain Serine Protease family B	extracellular region	hydrolase activity;peptidase activity;serine-type endopeptidase activity;serine-type peptidase activity	proteolysis	carcass_7
AAEL006377	LRIM31	leucine-rich immune protein (Coil-less)	N/A	protein binding	N/A	carcass_4, carcass_7
AAEL006674	CLIPB29	Clip-Domain Serine Protease family B.	extracellular region	hydrolase activity;peptidase activity;serine-type endopeptidase activity;serine-type peptidase activity	proteolysis	carcass_4
AAEL007224	LRIM22	leucine-rich immune protein (Coil-less)	N/A	protein binding	N/A	carcass_4, carcass_7
AAEL007696	REL1A	TOLL pathway signalling NF-kappaB Relish-like transcription factor	cytoplasm;host cell nucleus;nucleus	DNA binding;DNA-binding transcription factor activity	regulation of transcription, DNA-templated	carcass_4
AAEL008370	SCRB17	Class B Scavenger Receptor (CD36 domain).	integral component of membrane;membrane	N/A	N/A	carcass_4
AAEL009178	GNBPB4	Gram-Negative Binding Protein (GNBP) or Beta-1 3-Glucan Binding Protein (BGBP).	N/A	hydrolase activity, hydrolyzing O-glycosyl compounds	carbohydrate metabolic process	carcass_7
AAEL009420	SCRBQ1	Class B Scavenger Receptor (CD36 domain).	integral component of membrane;membrane	N/A	N/A	carcass_7
AAEL009474	PGRPS1	Peptidoglycan Recognition Protein (Short)	N/A	N-acetylmuramoyl-L-alanine amidase activity;peptidoglycan binding;zinc ion binding	immune system process;innate immune response;peptidoglycan catabolic process	carcass_4
AAEL009842	GALE12	Galectin [Source:UniProtKB/TrEMBL;Acc:Q16UP1]	N/A	N/A	N/A	carcass_4
AAEL010128	LRIM4	leucine-rich immune protein (Long)	N/A	protein binding	N/A	carcass_4
AAEL011009	N/A	fibrinogen and fibronectin	N/A	N/A	N/A	carcass_4
AAEL011407	CTL20	C-Type Lectin (CTL20)	N/A	N/A	N/A	carcass_7

AAEL011408	CTL21	C-Type Lectin (CTL21)	N/A	N/A	N/A	carcass_4, carcass_7
AAEL011453	CTL14	C-Type Lectin (CTL14)	N/A	N/A	N/A	carcass_4, carcass_7
AAEL011455	CTLMA12	C-Type Lectin (CTLMA12) - mannose binding	N/A	carbohydrate binding	N/A	carcass_4
AAEL011621	CTLMA13	C-Type Lectin (CTL) - mannose binding.	N/A	carbohydrate binding;serine-type endopeptidase activity	proteolysis	carcass_4, carcass_7
AAEL011633	N/A	fibrinogen and fibronectin	N/A	N/A	N/A	carcass_4, carcass_7
AAEL011634	N/A	fibrinogen and fibronectin	N/A	N/A	N/A	carcass_7
AAEL011763	PPO3	prophenoloxidase	N/A	metal ion binding;oxidoreductase activity	obsolete oxidation-reduction process	carcass_4, carcass_7
AAEL011764	PPO10	prophenoloxidase	N/A	metal ion binding;oxidoreductase activity	obsolete oxidation-reduction process	carcass_4, carcass_7
AAEL012086	LRIM1	leucine-rich immune protein (Long)	N/A	protein binding	N/A	carcass_4
AAEL012135	GALE2	Galectin [Source:UniProtKB/TrEMBL;Acc:Q16MZ7]	N/A	carbohydrate binding	N/A	carcass_4
AAEL012255	LRIM13	leucine-rich immune protein (Short)	N/A	protein binding	N/A	midgut_7, carcass_4, carcass_7
AAEL012410	AGO1b	eukaryotic translation initiation factor 2C	N/A	nucleic acid binding;protein binding	N/A	midgut_4
AAEL012538	LRIM6	leucine-rich immune protein (Short)	N/A	protein binding	N/A	carcass_4, carcass_7
AAEL012767	LRIM5	leucine-rich immune protein (Short)	N/A	protein binding	N/A	carcass_4
AAEL013245	CLIPB28	Clip-Domain Serine Protease family B.	N/A	peptidase activity;serine-type endopeptidase activity	proteolysis	carcass_4, carcass_4
AAEL013417	N/A	fibrinogen and fibronectin	N/A	N/A	N/A	carcass_7
AAEL013434	N/A	spaetzle-like cytokine	N/A	N/A	N/A	carcass_7
AAEL013496	PPO8	prophenoloxidase	N/A	metal ion binding;oxidoreductase activity	obsolete oxidation-reduction process	carcass_4, carcass_7
AAEL013501	PPO4	prophenoloxidase	N/A	metal ion binding;oxidoreductase activity	obsolete oxidation-reduction process	carcass_4
AAEL014349	CLIPB15	Clip-Domain Serine Protease family B.	extracellular region	hydrolase activity;peptidase activity;serine-type endopeptidase activity;serine-type peptidase activity	proteolysis	carcass_4

AAEL014382	CTLMA14	C-Type Lectin (CTL) - mannose binding.	N/A	carbohydrate binding	N/A	carcass_4
AAEL017249	SRPN24	Serine Protease Inhibitor (serpin) homologue - unlikely to be inhibitory.	extracellular space	N/A	N/A	carcass_4
AAEL017536	GRRP	holotricin glycine rich repeat protein (GRRP) anti-microbial peptide	N/A	N/A	N/A	carcass_4, carcass_4, carcass_4, carcass_7, carcass_7

CC, cellular component; MF, molecular function; BP, biological process.
