# Supplementary material for: Wolbachia-mediated resistance to Zika virus infection in Aedes aegypti is dominated by diverse transcriptional regulation and weak evolutionary pressures
Source: PLoS Negl Trop Dis. 2023 Oct 2;17(10):e0011674. doi: 10.1371/journal.pntd.0011674 (PMC10569609; doi:10.1371/journal.pntd.0011674)
Supplement: S3 Table — (PDF) [file pntd.0011674.s010.pdf]

**S3 Table. Genes differentially expressed in ZIKV-exposed COL.*w*Mel carcasses 7dpf.**		
**Gene ID**	**Product Description**	**Gene Name or Symbol**
**AAEL000102**	unspecified product	N/A
**AAEL000311**	unspecified product	N/A
**AAEL000415**	AMP dependent coa ligase	N/A
**AAEL000566**	unspecified product	N/A
**AAEL000658**	unspecified product	N/A
**AAEL000859**	unspecified product	N/A
**AAEL001062**	unspecified product	N/A
**AAEL001091**	Malic enzyme [Source:UniProtKB/TrEMBL;Acc:A0A1S4EXR8]	N/A
**AAEL001209**	sodium-dependent phosphate transporter	N/A
**AAEL001232**	tubulointerstitial nephritis antigen	N/A
**AAEL001293**	unspecified product	N/A
**AAEL001307**	SEC14, putative	N/A
**AAEL001818**	unspecified product	N/A
**AAEL002378**	Carboxylic ester hydrolase [Source:UniProtKB/TrEMBL;Acc:A0A0P6IYI7]	N/A
**AAEL002416**	short-chain dehydrogenase	N/A
**AAEL002467**	unspecified product	N/A
**AAEL002796**	l-asparaginase i	N/A
**AAEL002978**	leucyl aminopeptidase, putative	N/A
**AAEL003002**	unspecified product	N/A
**AAEL003803**	unspecified product	N/A
**AAEL004297**	ATP-citrate synthase	N/A
**AAEL004941**	cytochrome P450	CYP6AK1
**AAEL004974**	beta-1,3-glucuronyltransferase s, p	N/A
**AAEL005147**	unspecified product	N/A
**AAEL005199**	Carboxylic ester hydrolase [Source:UniProtKB/TrEMBL;Acc:A0A1S4F9U8]	N/A
**AAEL005256**	unspecified product	N/A
**AAEL005293**	Galectin [Source:UniProtKB/TrEMBL;Acc:Q16ND5]	GALE8A
**AAEL005428**	unspecified product	N/A
**AAEL005515**	heterogeneous nuclear ribonucleoprotein	N/A
**AAEL005790**	malic enzyme	N/A
**AAEL005992**	adam (a disintegrin and metalloprotease)	N/A
**AAEL006721**	2-oxoglutarate dehydrogenase	N/A
**AAEL006883**	unspecified product	N/A
**AAEL007010**	cytochrome P450	CYP6AG4
**AAEL007029**	tropomodulin	N/A
**AAEL007271**	basic helix-loop-helix zip transcription factor	N/A
**AAEL007381**	unspecified product	N/A
**AAEL007653**	allantoinase	N/A
**AAEL007914**	discs large protein	N/A

**AAEL009129**	cytochrome P450	CYP6Z9
**AAEL009630**	high-affinity cgmp-specific 3,5-cyclic phosphodiesterase	N/A
**AAEL009645**	unspecified product	N/A
**AAEL010075**	oxidoreductase	N/A
**AAEL010128**	leucine-rich immune protein (Long)	LRIM4
**AAEL010366**	glucosyl/glucuronosyl transferases	N/A
**AAEL010712**	low-density lipoprotein receptor (ldl)	N/A
**AAEL011006**	guanylate kinase	N/A
**AAEL011133**	unspecified product	N/A
**AAEL011161**	unspecified product	N/A
**AAEL012110**	protease m1 zinc metalloprotease	N/A
**AAEL012409**	pantothenate kinase	N/A
**AAEL012446**	Inhibitor of Apoptosis (IAP) containing Baculoviral IAP Repeat(s) (BIR domains).	IAP6
**AAEL012740**	ATPase subunit, putative	N/A
**AAEL013262**	unspecified product	N/A
**AAEL013347**	lethal(2)essential for life protein, l2efl	N/A
**AAEL013349**	lethal(2)essential for life protein, l2efl	N/A
**AAEL013431**	proline oxidase	N/A
**AAEL013484**	unspecified product	N/A
**AAEL013662**	anterior fat body protein	N/A
**AAEL013812**	unspecified product	N/A
**AAEL013885**	unspecified product	N/A
**AAEL014303**	neuroligin,	N/A
**AAEL014578**	ssm4 protein	N/A
**AAEL014619**	cytochrome P450	CYP9J22
**AAEL014863**	glycogenin	N/A
**AAEL014999**	unspecified product	N/A
**AAEL017514**	unspecified product	N/A
**AAEL018117**	unspecified product	N/A
**AAEL018219**	unspecified product	N/A
**AAEL018668**	ATP synthase F0 subunit 6	ATP6
**AAEL018685**	cytochrome b	CYTB
**AAEL019494**	unspecified product	N/A
**AAEL019495**	unspecified product	N/A
**AAEL019504**	unspecified product	N/A
**AAEL019639**	unspecified product	N/A
**AAEL019713**	unspecified product	N/A
**AAEL020524**	unspecified product	N/A
**AAEL020997**	pseudogene	N/A
**AAEL021035**	unspecified product	N/A
**AAEL021471**	unspecified product	N/A
**AAEL021762**	unspecified product	N/A
**AAEL021861**	unspecified product	N/A
**AAEL022059**	pseudogene	N/A

**AAEL023634**	unspecified product	N/A
**AAEL023799**	pseudogene	N/A
**AAEL024512**	pseudogene	N/A
**AAEL025488**	unspecified product	N/A
**AAEL027008**	unspecified product	N/A
**AAEL027243**	pseudogene	N/A
**AAEL027593**	unspecified product	N/A
**AAEL027694**	unspecified product	N/A
**AAEL028635**	unspecified product	N/A
**AAEL029056**	unspecified product	N/A
