# Supplementary material for: Wolbachia-mediated resistance to Zika virus infection in Aedes aegypti is dominated by diverse transcriptional regulation and weak evolutionary pressures
Source: PLoS Negl Trop Dis. 2023 Oct 2;17(10):e0011674. doi: 10.1371/journal.pntd.0011674 (PMC10569609; doi:10.1371/journal.pntd.0011674)
Supplement: S4 Table — (PDF) [file pntd.0011674.s011.pdf]

S4 Table. Genes differentially expressed in ZIKV-exposed COL.*w*Mel midguts 7dpf.		
**Gene ID**	**Product Description**	**Gene Name or Symbol**
**AAEL000080**	phosphoenolpyruvate carboxykinase	N/A
**AAEL000128**	P130	N/A
**AAEL000271**	gamma-glutamyl hydrolase	N/A
**AAEL000294**	unspecified product	N/A
**AAEL000323**	cysteine-rich venom protein, putative	N/A
**AAEL000416**	FHA domain-containing protein [Source:UniProtKB/TrEMBL;Acc:A0A1S4EVV1]	N/A
**AAEL000488**	unspecified product	N/A
**AAEL000512**	Dynein heavy chain [Source:UniProtKB/TrEMBL;Acc:A0A1S4EW29]	N/A
**AAEL000566**	unspecified product	N/A
**AAEL000636**	unspecified product	N/A
**AAEL000713**	reticulon/nogo	N/A
**AAEL000757**	anterior fat body protein	N/A
**AAEL000859**	unspecified product	N/A
**AAEL000898**	unspecified product	N/A
**AAEL000902**	sugar transporter	N/A
**AAEL000905**	unspecified product	N/A
**AAEL000923**	unspecified product	N/A
**AAEL001062**	unspecified product	N/A
**AAEL001209**	sodium-dependent phosphate transporter	N/A
**AAEL001232**	tubulointerstitial nephritis antigen	N/A
**AAEL001254**	unspecified product	N/A
**AAEL001421**	high density lipoprotien binding protein / vigilin	N/A
**AAEL001434**	coronin	N/A
**AAEL001511**	unspecified product	N/A
**AAEL001532**	FAD NAD binding oxidoreductases	N/A
**AAEL001580**	otefin, putative	N/A
**AAEL001632**	multicopper oxidase	N/A
**AAEL001650**	ML domain-containing protein [Source:UniProtKB/TrEMBL;Acc:A0A1S4EZF1]	N/A
**AAEL001667**	multicopper oxidase	N/A
**AAEL001749**	ventrhoid transmembrane protein, putative	N/A
**AAEL001816**	glucosyl/glucuronosyl transferases	N/A
**AAEL001818**	unspecified product	N/A
**AAEL001837**	Lipase [Source:UniProtKB/TrEMBL;Acc:A0A1S4EZX5]	N/A

**AAEL001900**	lactosylceramide 4-alpha-galactosyltransferase (alpha-1,4-galactosyltransferase)	N/A
**AAEL001905**	unspecified product	N/A
**AAEL001919**	protein tyrosine phosphatase, non-receptor type nt1	N/A
**AAEL001986**	kinesin-like protein KIF1B	N/A
**AAEL002036**	unspecified product	N/A
**AAEL002080**	septin interacting protein, putative	N/A
**AAEL002102**	unspecified product	N/A
**AAEL002109**	unspecified product	N/A
**AAEL002130**	ecdysone inducible protein L2, putative	N/A
**AAEL002176**	inosine-uridine preferring nucleoside hydrolase	N/A
**AAEL002199**	unspecified product	N/A
**AAEL002235**	unspecified product	N/A
**AAEL002261**	GTP cyclohydrolase i	N/A
**AAEL002309**	Thioredoxin Peroxidase.	TPX4
**AAEL002467**	unspecified product	N/A
**AAEL002557**	cationic amino acid transporter	N/A
**AAEL002623**	unspecified product	N/A
**AAEL002652**	unspecified product	N/A
**AAEL002661**	Matrix metalloproteinase [Source:UniProtKB/TrEMBL;Acc:A0A1S4F2E2]	N/A
**AAEL002671**	unspecified product	N/A
**AAEL002690**	beat protein	N/A
**AAEL002714**	kinesin-like protein KIF23 (mitotic kinesin-like protein 1)	N/A
**AAEL002757**	unspecified product	N/A
**AAEL002796**	l-asparaginase i	N/A
**AAEL002848**	tubulin beta chain	N/A
**AAEL002854**	F-box domain-containing protein [Source:UniProtKB/TrEMBL;Acc:A0A1S4F302]	N/A
**AAEL002886**	thioredoxin reductase	N/A
**AAEL002919**	unspecified product	N/A
**AAEL002921**	unspecified product	N/A
**AAEL002978**	leucyl aminopeptidase, putative	N/A
**AAEL003051**	unspecified product	N/A
**AAEL003063**	Semaphorin [Source:UniProtKB/TrEMBL;Acc:A0A1S4F3R5]	N/A
**AAEL003294**	fibrinogen and fibronectin	N/A
**AAEL003317**	alkaline phosphatase	N/A
**AAEL003589**	transcription factor, putative	N/A
**AAEL003619**	sodium/chloride dependent amino acid transporter	N/A

**AAEL003681**	unspecified product	N/A
**AAEL003712**	C-Type Lysozyme (Lys-E).	LYSC10
**AAEL003950**	helicase	N/A
**AAEL003951**	unspecified product	N/A
**AAEL004090**	unspecified product	N/A
**AAEL004092**	deoxyribonuclease I, putative	N/A
**AAEL004126**	sterol desaturase	N/A
**AAEL004206**	unspecified product	N/A
**AAEL004212**	unspecified product	N/A
**AAEL004302**	unspecified product	N/A
**AAEL004310**	p15-2a protein, putative	N/A
**AAEL004319**	epidermal growth factor receptor	N/A
**AAEL004386**	chorion peroxidase	pxt
**AAEL004392**	IAP-antagonist Michelob_x-like Protein	IMP
**AAEL004520**	cAMP/cgmp cyclic nucleotide phosphodiesterase	N/A
**AAEL004710**	spingomyelin synthetase	N/A
**AAEL004729**	unspecified product	N/A
**AAEL004868**	hemomucin	N/A
**AAEL004870**	cytochrome P450	CYP18A1
**AAEL004964**	unspecified product	N/A
**AAEL004981**	cation-transporting ATPase	N/A
**AAEL005008**	aquaporin	N/A
**AAEL005071**	GTP binding protein [Source:UniProtKB/TrEMBL;Acc:A0A1S4F9G8]	N/A
**AAEL005255**	PAR-domain protein 1	PDP1
**AAEL005342**	unspecified product	N/A
**AAEL005347**	unspecified product	N/A
**AAEL005417**	annexin x	N/A
**AAEL005428**	unspecified product	N/A
**AAEL005432**	unspecified product	N/A
**AAEL005455**	CTP synthase [Source:UniProtKB/TrEMBL;Acc:Q17A05]	CTPsyn
**AAEL005503**	unspecified product	N/A
**AAEL005666**	matrix metalloproteinase	N/A
**AAEL005701**	retinaldehyde binding protein	N/A
**AAEL005704**	unspecified product	N/A
**AAEL005791**	unspecified product	N/A
**AAEL005839**	uridine phosphorylase	N/A
**AAEL005977**	chondroitin 4-sulfotransferase	N/A

**AAEL005992**	adam (a disintegrin and metalloprotease)	N/A
**AAEL006028**	unspecified product	N/A
**AAEL006034**	Vanin-like protein 1 precursor, putative	N/A
**AAEL006054**	peptidyl-prolyl cis-trans isomerase (cyclophilin)	N/A
**AAEL006171**	n-myc downstream regulated	N/A
**AAEL006216**	unspecified product	N/A
**AAEL006277**	unspecified product	N/A
**AAEL006321**	1-acylglycerol-3-phosphate acyltransferase [Source:UniProtKB/TrEMBL;Acc:Q176M5]	N/A
**AAEL006355**	Class C Scavenger Receptor (Sushi/SCR/CCP MAM and Somatomedin B domains).	SCRC1
**AAEL006361**	Class C Scavenger Receptor (Sushi/SCR/CCP MAM and Somatomedin B domains).	SCRC2
**AAEL006449**	ser/thr protein kinase-lyk4	N/A
**AAEL006480**	unspecified product	N/A
**AAEL006518**	cytidine deaminase, putative	N/A
**AAEL006663**	ANK_REP_REGION domain-containing protein [Source:UniProtKB/TrEMBL;Acc:A0A1S4FEA3]	N/A
**AAEL006686**	unspecified product	N/A
**AAEL006708**	hedgehog	N/A
**AAEL006723**	unspecified product	N/A
**AAEL006809**	voltage-gated ion channel	N/A
**AAEL006902**	serine-type enodpeptidase,	N/A
**AAEL006921**	calmodulin	N/A
**AAEL006978**	protein-glutamine gamma-glutamyltransferase	N/A
**AAEL007004**	GPCR Bride of Sevenless Family	GPRBOS1
**AAEL007018**	udp-glucose 4-epimerase	N/A
**AAEL007030**	ceramidase	N/A
**AAEL007097**	4-nitrophenylphosphatase [Source:UniProtKB/TrEMBL;Acc:Q0IF18]	N/A
**AAEL007120**	lim homeobox protein	N/A
**AAEL007191**	amino acid transporter	N/A
**AAEL007208**	unspecified product	N/A
**AAEL007238**	DUF3421 domain-containing protein [Source:UniProtKB/TrEMBL;Acc:A0A1S4FG14]	N/A
**AAEL007258**	unspecified product	N/A
**AAEL007271**	basic helix-loop-helix zip transcription factor	N/A
**AAEL007299**	Cadherin, putative [Source:UniProtKB/TrEMBL;Acc:A0A1S4FG21]	N/A
**AAEL007344**	LITAF domain-containing protein [Source:UniProtKB/TrEMBL;Acc:A0A1S4FG64]	N/A
**AAEL007547**	chloride channel protein	N/A

**AAEL007560**	core 1 udp-galactose:n-acetylgalactosamine-alpha-r beta 1,3-galactosyltransferase	N/A
**AAEL007657**	low-density lipoprotein receptor (ldl)	N/A
**AAEL007765**	Serine Protease Inhibitor (serpin) likely cleavage at K/R. Transcript A.	SRPN10
**AAEL007778**	leucine-rich transmembrane protein	N/A
**AAEL007872**	unspecified product	N/A
**AAEL007880**	ornithine decarboxylase	N/A
**AAEL007942**	fibrinogen and fibronectin	N/A
**AAEL007993**	Clip-Domain Serine Protease family B.	CLIPB27
**AAEL008024**	unspecified product	N/A
**AAEL008027**	unspecified product	N/A
**AAEL008028**	monocarboxylate transporter	N/A
**AAEL008097**	trypsin-eta, putative	N/A
**AAEL008141**	period circadian protein	PER
**AAEL008267**	GPCR Neurokinin/Tachykinin Family	GPRNPR5
**AAEL008306**	mitogen activated protein kinase kinase kinase 5, mapkkk5, mekk5	N/A
**AAEL008346**	achaete-scute complex protein T3, putative	N/A
**AAEL008467**	cysteine synthase	N/A
**AAEL008468**	cysteine synthase	N/A
**AAEL008473**	cysteine-rich venom protein, putative	N/A
**AAEL008511**	unspecified product	N/A
**AAEL008547**	unspecified product	N/A
**AAEL008622**	jnk	N/A
**AAEL008655**	GPCR Vasopressin Family	GPRVPR2
**AAEL008658**	leucine-rich immune protein (TM)	LRIM16
**AAEL008760**	unspecified product	N/A
**AAEL008767**	serine protease	N/A
**AAEL008782**	serine-type enodpeptidase,	N/A
**AAEL008829**	unspecified product	N/A
**AAEL008832**	forkhead box protein (AaegFOXN1)	N/A
**AAEL008843**	unspecified product	N/A
**AAEL008910**	unspecified product	N/A
**AAEL008916**	unspecified product	N/A
**AAEL008921**	myosin regulatory light chain 2 smooth muscle	N/A
**AAEL008953**	unspecified product	N/A
**AAEL009070**	unspecified product	N/A
**AAEL009114**	unspecified product	N/A
**AAEL009185**	arginine or creatine kinase	N/A

**AAEL009249**	Coronin [Source:UniProtKB/TrEMBL;Acc:A0A1S4FM51]	N/A
**AAEL009317**	rab11	N/A
**AAEL009333**	unspecified product	N/A
**AAEL009371**	unspecified product	N/A
**AAEL009556**	Niemann-Pick Type C-2, putative	N/A
**AAEL009645**	unspecified product	N/A
**AAEL009681**	Putative rhomboid family [Source:UniProtKB/TrEMBL;Acc:A0A0P6IZ43]	N/A
**AAEL009762**	cytochrome P450	CYP307A1
**AAEL009813**	glutamate receptor 7 (ampa)	N/A
**AAEL009842**	Galectin [Source:UniProtKB/TrEMBL;Acc:Q16UP1]	GALE12
**AAEL009850**	Galectin [Source:UniProtKB/TrEMBL;Acc:Q16UP0]	GALE14
**AAEL009987**	unspecified product	N/A
**AAEL010050**	unspecified product	N/A
**AAEL010075**	oxidoreductase	N/A
**AAEL010084**	unspecified product	N/A
**AAEL010094**	cyclin b	N/A
**AAEL010145**	sodium/potassium-dependent ATPase beta-2 subunit	N/A
**AAEL010264**	unspecified product	N/A
**AAEL010270**	unspecified product	N/A
**AAEL010375**	unspecified product	N/A
**AAEL010477**	unspecified product	N/A
**AAEL010483**	oxysterol-binding protein related protein (ORP8)	ORP8
**AAEL010650**	sodium/solute symporter	N/A
**AAEL010661**	phospholipid scramblase 1,	N/A
**AAEL010678**	unspecified product	N/A
**AAEL010738**	sodium bicarbonate cotransporter	N/A
**AAEL010776**	carboxypeptidase	N/A
**AAEL010782**	carboxypeptidase	N/A
**AAEL010840**	unspecified product	N/A
**AAEL010932**	RNAse h	N/A
**AAEL010956**	unspecified product	N/A
**AAEL011009**	fibrinogen and fibronectin	N/A
**AAEL011203**	unspecified product	N/A
**AAEL011264**	phosphatidylethanolamine-binding protein	N/A
**AAEL011424**	Histone H3 [Source:UniProtKB/TrEMBL;Acc:A0A1S4FTJ0]	N/A
**AAEL011510**	multiple inositol polyphosphate phosphatase	N/A
**AAEL011598**	Gustatory receptor [Source:UniProtKB/TrEMBL;Acc:A0A1S4FU22]	N/A

**AAEL011648**	cyclin d	N/A
**AAEL011650**	coatomer, gamma-subunit, putative	N/A
**AAEL011653**	thyroid hormone receptor interactor	N/A
**AAEL011901**	1-acyl-sn-glycerol-3-phosphate acyltransferase	N/A
**AAEL011937**	glucosyl/glucuronosyl transferases	N/A
**AAEL012003**	Galectin [Source:UniProtKB/TrEMBL;Acc:Q17AG2]	GALE6B
**AAEL012014**	L-lactate dehydrogenase [Source:UniProtKB/TrEMBL;Acc:Q16ND1]	N/A
**AAEL012052**	unspecified product	N/A
**AAEL012062**	Na+/K+ ATPase alpha subunit	N/A
**AAEL012349**	lipase 1 precursor	N/A
**AAEL012390**	unspecified product	N/A
**AAEL012410**	eukaryotic translation initiation factor 2C	AGO1b
**AAEL012499**	Histone H2A [Source:UniProtKB/TrEMBL;Acc:Q16LW9]	N/A
**AAEL012514**	translation initiation factor 2b, delta subunit	N/A
**AAEL012522**	Sodium-dependent phosphate transporter [Source:UniProtKB/TrEMBL;Acc:A0A1S4FWV3]	N/A
**AAEL012545**	Proliferating cell nuclear antigen [Source:UniProtKB/TrEMBL;Acc:Q4PKD7]	N/A
**AAEL012629**	deoxyuridine 5'-triphosphate nucleotidohydrolase	N/A
**AAEL012859**	unspecified product	N/A
**AAEL012960**	importin alpha	N/A
**AAEL013111**	glutamate transporter	N/A
**AAEL013262**	unspecified product	N/A
**AAEL013276**	acid phosphatase	N/A
**AAEL013304**	unspecified product	N/A
**AAEL013309**	high-affinity copper uptake protein	N/A
**AAEL013345**	alphaA-crystallin, putative	N/A
**AAEL013346**	lethal(2)essential for life protein, l2efl	N/A
**AAEL013348**	lethal(2)essential for life protein, l2efl	N/A
**AAEL013349**	lethal(2)essential for life protein, l2efl	N/A
**AAEL013350**	heat shock protein 26kD, putative	N/A
**AAEL013351**	lethal(2)essential for life protein, l2efl	N/A
**AAEL013352**	lethal(2)essential for life protein, l2efl	N/A
**AAEL013692**	PIWI	PIWI3
**AAEL013713**	trypsin	N/A
**AAEL013780**	unspecified product	N/A
**AAEL013808**	fascin	N/A
**AAEL013875**	tetraspanin, putative	N/A
**AAEL014019**	cytochrome P450	CYP4J16

**AAEL014226**	unspecified product	N/A
**AAEL014251**	Inhibitor of Apoptosis (IAP) containing Baculoviral IAP Repeat(s) (BIR domains).	IAP5
**AAEL014348**	caspase (short)	CASPS8
**AAEL014363**	unspecified product	N/A
**AAEL014408**	m-phase inducer phosphatase(cdc25)	N/A
**AAEL014439**	juvenile hormone-inducible protein, putative	N/A
**AAEL014454**	unspecified product	N/A
**AAEL014541**	maltose phosphorylase	N/A
**AAEL014567**	oviductin	N/A
**AAEL014945**	unspecified product	N/A
**AAEL014981**	unspecified product	N/A
**AAEL016975**	unspecified product	N/A
**AAEL017331**	unspecified product	N/A
**AAEL017553**	Carboxy/choline esterase Alpha Esterase	CCEAE2B
**AAEL018039**	unspecified product	N/A
**AAEL018118**	unspecified product	N/A
**AAEL018120**	Ribosomal protein S6 kinase [Source:UniProtKB/TrEMBL;Acc:Q535V4]	N/A
**AAEL019504**	unspecified product	N/A
**AAEL019536**	unspecified product	N/A
**AAEL019604**	unspecified product	N/A
**AAEL019610**	unspecified product	N/A
**AAEL019623**	unspecified product	N/A
**AAEL019677**	unspecified product	N/A
**AAEL019681**	unspecified product	N/A
**AAEL019700**	unspecified product	N/A
**AAEL019712**	unspecified product	N/A
**AAEL019722**	unspecified product	N/A
**AAEL019728**	suppressor of cytokine signaling	SOCS
**AAEL019785**	unspecified product	N/A
**AAEL019793**	unspecified product	N/A
**AAEL019902**	unspecified product	N/A
**AAEL019903**	unspecified product	N/A
**AAEL019940**	unspecified product	N/A
**AAEL019941**	unspecified product	N/A
**AAEL020654**	unspecified product	N/A
**AAEL020729**	unspecified product	N/A
**AAEL020800**	unspecified product	N/A

**AAEL021278**	unspecified product	N/A
**AAEL021318**	unspecified product	N/A
**AAEL021321**	unspecified product	N/A
**AAEL021899**	unspecified product	N/A
**AAEL021925**	unspecified product	N/A
**AAEL021931**	unspecified product	N/A
**AAEL022048**	unspecified product	N/A
**AAEL022059**	pseudogene	N/A
**AAEL022167**	unspecified product	N/A
**AAEL022628**	unspecified product	N/A
**AAEL022659**	unspecified product	N/A
**AAEL023231**	unspecified product	N/A
**AAEL023478**	unspecified product	N/A
**AAEL023490**	unspecified product	N/A
**AAEL023644**	unspecified product	N/A
**AAEL023746**	unspecified product	N/A
**AAEL024003**	unspecified product	N/A
**AAEL024222**	unspecified product	N/A
**AAEL024370**	unspecified product	N/A
**AAEL024512**	pseudogene	N/A
**AAEL024520**	unspecified product	N/A
**AAEL024558**	unspecified product	N/A
**AAEL024675**	unspecified product	N/A
**AAEL024880**	unspecified product	N/A
**AAEL024913**	unspecified product	N/A
**AAEL024940**	unspecified product	N/A
**AAEL025091**	unspecified product	N/A
**AAEL025226**	unspecified product	N/A
**AAEL025432**	Cytidine deaminase [Source:UniProtKB/TrEMBL;Acc:Q0IF52]	N/A
**AAEL025530**	unspecified product	N/A
**AAEL025658**	unspecified product	N/A
**AAEL025718**	unspecified product	N/A
**AAEL025818**	unspecified product	N/A
**AAEL025839**	unspecified product	N/A
**AAEL026025**	unspecified product	N/A
**AAEL026215**	unspecified product	N/A
**AAEL026343**	unspecified product	N/A

**AAEL026466**	unspecified product	N/A
**AAEL026744**	unspecified product	N/A
**AAEL026751**	unspecified product	N/A
**AAEL026981**	unspecified product	N/A
**AAEL027019**	unspecified product	N/A
**AAEL027398**	unspecified product	N/A
**AAEL027593**	unspecified product	N/A
**AAEL027610**	unspecified product	N/A
**AAEL027937**	unspecified product	N/A
**AAEL028005**	unspecified product	N/A
**AAEL028021**	unspecified product	N/A
**AAEL028048**	unspecified product	N/A
**AAEL028088**	unspecified product	N/A
**AAEL028635**	unspecified product	N/A
**AAEL029041**	unspecified product	N/A
**AAEL029044**	cecropin	CECE
**AAEL029046**	cecropin	CECD
**AAEL029082**	unspecified product	N/A
**AAEL029104**	unspecified product	N/A
**AAEL029107**	unspecified product	N/A
